# Microbial Volatile Organic Compounds from Tempered and Incubated Grain Mediate Attraction by a Primary but Not Secondary Stored Product Insect Pest in Wheat

**DOI:** 10.1007/s10886-021-01312-8

**Published:** 2021-09-20

**Authors:** Taylor Van Winkle, Marco Ponce, Hannah Quellhorst, Alexander Bruce, Chloe E. Albin, Tania N. Kim, Kun Yan Zhu, William R. Morrison

**Affiliations:** 1 School of Planning, Design, and Construction, Michigan State University, East Lansing, MI USA; 2 Department of Entomology, Kansas State University, Manhattan, KS 66506 USA; 3 USDA-ARS Center for Grain and Animal Health Research, Manhattan, KS 66502 USA; 4 Department of Engineering, Kansas State University, Manhattan, KS 66506 USA

**Keywords:** Postharvest, Taxis, Microbial cues, *Rhyzopertha dominica*, *Tribolium castaneum*, Insect-microbe interactions

## Abstract

There has been a dearth of research elucidating the behavioral effect of microbially-produced volatile organic compounds on insects in postharvest agriculture. Demonstrating attraction to MVOC’s by stored product insects would provide an additional source of unique behaviorally-relevant stimuli to protect postharvest commodities at food facilities. Here, we assessed the behavioral response of a primary (*Rhyzopertha dominica*) and secondary (*Tribolium castaneum*) grain pest to bouquets of volatiles produced by whole wheat that were untempered, or tempered to 12%, 15%, or 19% grain moisture and incubated for 9, 18, or 27 days. We hypothesized that MVOC’s may be more important for the secondary feeder because they signal that otherwise unusable, intact grains have become susceptible by weakening of the bran. However, contrary to our expectations, we found that the primary feeder, *R. dominica*, but not *T. castaneum* was attracted to MVOC’s in a wind tunnel experiment, and in a release-recapture assay using commercial traps baited with grain treatments. Increasing grain moisture resulted in elevated grain damage detected by near-infrared spectroscopy and resulted in small but significant differences in the blend of volatiles emitted by treatments detected by gas chromatography coupled with mass spectrometry (GC–MS). In sequencing the microbial community on the grain, we found a diversity of fungi, suggesting that an assemblage was responsible for emissions. We conclude that *R. dominica* is attracted to a broader suite of MVOC’s than *T. castaneum*, and that our work highlights the importance of understanding insect-microbe interactions in the postharvest agricultural supply chain.

**Supplementary Information:**

The online version contains supplementary material available at 10.1007/s10886-021-01312-8.

## Introduction

Olfaction mediates many fundamental biological processes for insects. For example, volatile compounds may be used for foraging (Morrison et al. 2018a), mate-finding (Xu and Turlings 2018), development (Oi et al. 2015), dispersal (Rork and Renner 2018), as well as social group formation and cohesion (Buhl and Rogers 2016). These compounds may originate from a variety of sources, including plant volatiles (Xu and Turlings 2018), microbes (e.g., fungi, bacteria; Davis et al. 2013), conspecifics, and heterospecifics.

There have now been an increasing number of studies in a variety of systems exploring insect-microbe interactions, and how microbially-produced volatile organic compounds (MVOC’s) significantly alter ecological interactions (Bueno et al. 2020; Liu et al. 2016; Russell and Ashman 2019). For instance, it is known that MVOC’s may aggregate insects, attract individuals, promote mating or oviposition, elicit feeding, or act as strong repellents (reviewed in Davis et al. 2013). The behavioral response to the same microorganism or MVOC may vary by species or abiotic conditions (Hoftstetter et al. 2007). Further, MVOC’s may signal host suitability and may even indicate a specific timeframe for optimal conditions to insects (Beck and Vannette 2017). Davis and Landolt (2013) found that over 1,300 insects, representing 39 species, responded to three common fungal volatiles, 2-methyl-1-butanol, 3-methyl-1-butanol, and 2-phenylethanol, found in the headspace of the yeast-like fungus *Aureobasidium pullulans* on a spearmint farm. The authors concluded that the insects were not responding haphazardly, because of the low insect attraction to traps with competing volatiles from another species of fungus. In some cases, insects will respond similarly to microbial assemblages as to microbial monocultures (Rering et al. 2020). Thus, in the last decade, there has been an increasing amount of work documenting the ubiquity and importance of MVOC’s for insect behavioral responses (Beck et al. 2018; Davis et al. 2013; Ponce et al. 2021; Weisskopf et al. 2021).

In evolutionary terms, stored product insects have only relatively recently switched to feeding on agricultural goods (e.g., in the last 10,000 years bp). For most of their history, stored product insects were well-adapted for dispersing to, finding, and utilizing woody hosts (reviewed in Quellhorst et al. 2021) or animal caches of stored food (Jia et al. 2008). Animals typically store their food in damp conditions, often near the surface but underground or else in a concealed location (Hagstrum and Phillips 2017), which is an ideal environment for the growth of fungi and other microbes. Only about 60% of the caches were recovered by pine chipmunks in Nevada, for example (Kamil and Gould 2008), suggesting that at least 40% of their seed caches, but perhaps all of them, would be available for exploitation by stored product insects, depending on length of storage. Possible kairomones of that food source for stored product insects may be MVOC’s produced through colonization of and selective growth given the environmental conditions in animal caches by microbes (Morrison et al. 2021). Thus, there may be a conserved response by stored product insects to MVOC’s.

Indeed, attraction to MVOC’s by stored product insects has been observed in a variety of studies. For example, in a two-choice olfactometer, research demonstrated attraction of four species of stored product cucujid beetles to specific fungal volatiles, including 1-octen-3-one, 3-octanol, and 3-octanone (Pierce et al. 1991). Another study found that *Tribolium castaneum* (Herbst) (Coleoptera: Tenebrionidae) were more strongly attracted to fungi associated with cotton seed than those found on certain grains (Ahmad et al. 2012). Similarly, uncrowded or moderately crowded *Oryzaephilus surinamensis* (L.) (Coleoptera: Cucujidae) and *O. mercator* (Fauvel) (Coleoptera: Cucujidae) were highly attracted to volatiles from Brewer’s yeast (Pierce et al. 1983). By contrast, Zunino et al. (2015) found that eight MVOC’s produced by *Fusarium verticillioides* were repellent to the maize weevil, *Sitophilus zeamais* (Motschulsky) (Coleoptera: Cucurlionidae). Furthermore, other work found that stored-product pscocids were also repelled by volatiles produced by *Ulocladium botrytis* and *Eurotium amstelodami*, fungal strains isolated from decaying paper and colony food sources, respectively (Green 2008). In a systematic review, Ponce et al. (2021) found stored product arthropods were both attracted to and repelled by microbial cues, depending on arthropod species, microbial taxon, and other factors. Thus, it seems clear that MVOC’s are important for mediating behavioral responses for a range of stored product insects. While it may be impossible to work with sterile grain at food facilities, there has been an enormous amount of detail paid to hygiene in every process, including minimizing the occurrence of off-odor grain from microbial growth by creating conditions that are hostile for microbial proliferation. Thus, in modern food facilities, there is likely overall a dearth of MVOC’s because many managers of food facilities attempt to keep production areas well-sanitized (Morrison et al. 2019a, b; Morrison et al. 2021). As a result, MVOC’s represent at once both a potentially highly potent source of volatiles, and also unique cues in the post-harvest environment that is otherwise saturated with food stimuli. This potentially makes MVOC’s ideal to deploy against stored product insects to improve behaviorally-based monitoring and management tactics in food facilities. In particular, these cues may be useful in perimeter-based, attract-and-kill systems (Morrison et al. 2019a, b), push–pull systems that include a repellent (Pickett et al. 2014), or through the use of interception and spillage traps that include additional semiochemicals (Wilkins et al. 2021).

The microbial community for commodities in the field may be significantly different from that found after harvest during storage with an attendant change in key volatiles. For example, in sampling wheat heads in the field, the most common genera of fungi were *Alternaria*, *Cladosporium*, *Sporobolomyces*, *Blumeria*, and *Cryptoccus*, which together accounted for 83% of the species (Hertz et al. 2016). By contrast, the most abundant genera in storage on durum wheat were *Alternaria*, *Fusarium*, *Penicillium*, *Aspergillus*, *Rhizopus*, and *Mucor*, which together accounted for 96% of the species (Belkacem-Hanfi et al. 2013). While a systematic review found that eight-carbon compounds were common fungal volatiles in headspace (Ponce et al. 2021), no eight-carbon compounds were found emitted from a winter wheat field (Bachy et al. 2020), suggesting that in some cases the volatile composition in the field may vary greatly from microbial volatiles in storage. Thus, the food storage environment is a unique environment with its own biota and ecological dynamics worthy of investigation in its own right.

*Tribolium castaneum* and *Rhyzopertha dominica* (F.) (Coleoptera: Bostrichidae) are two important species in the food storage environment, and represent two different ends of a life history continuum for many variables, which may shed light on how life history shapes the use of fungal volatiles in foraging. While the former is a secondary pest, attacking finished or already damaged goods, *R. dominica* is a primary pest, seeking out intact grain kernels in which to oviposit and develop (Perišic et al. 2017). In terms of movement, *T. castaneum* is a strong walker but weak flier, while the opposite is true of *R. dominica* (Morrison et al. 2018b). Whereas *T. castaneum* is typically found associated with food facilities (Ridley et al. 2011), *R. dominica* can be found in high abundance elsewhere in the landscape far from food facilities (Mahroof et al. 2010). *Tribolium castaneum* has been described as facultatively frugivorous, with demonstrated attraction at small spatial scales to fungal volatiles from linted cotton (Ahmad et al. 2013), whereas there has been no study yet on the behavioral response of *R. dominica* to MVOC’s.

It is well-known that grain moisture affects a variety of parameters important for postharvest commodity protection. For example, most obviously, grain moisture may readily affect commodity quality (reviewed in Ziegler et al. 2021), and this is a parameter that is strictly monitored in food facilities. Generally, food facilities are xeric (very dry) environments, and some stored product insects have adapted to these conditions with the ability to make their own metabolic water in place of taking in water externally (Murdock et al. 2012). However, hotspots of insect infestation result in excess moisture in the commodity, which is absorbed by the grain, and may promote the growth of fungi and other microbes present on the commodity (Dunkel 1988). In addition, fungal growth in a xeric environment may be slow, but inexorable, and storage over increasing periods of time may lead to microbial spoilage. As a result, both grain moisture and storage interval are important determinants of microbial activity on grain.

The goal of the study was to evaluate the behavioral responses of *T. castaneum* and *R. dominica* to the MVOC’s produced by stored grain. We hypothesized that MVOC’s may be more important for the secondary feeder, *T. castaneum*, because MVOC’s may signal that otherwise unusable intact grains have become susceptible by weakening of the kernel through fungal activity. This may allow the secondary pest to more easily penetrate the bran of a kernel. We investigated the effects of grain moisture and incubation period on insect behavioral responses, and we also characterized changes in the volatile emissions caused by these variables.

## Methods and Materials

### Experimental Insects and Colony Maintenance

*Tribolium castaneum* were from a colony derived from a field population from central Kansas in 2012 and were reared on a mixture of 95% unbleached, organic flour and 5% brewer’s yeast. *Rhyzopertha dominica* were from a colony derived from a field population collected outside a mill in central Kansas in 2012 and were reared on organic wheat. Both colonies were maintained in an environmental chamber set at constant 27.5 °C, 60% RH, and 14:10 h (L:D) photoperiod.

For each assay, 4–8 wk-old adult were used. All beetles (in a 1:1 male:female sex ratio) for the assays were pulled from the colonies on the same day as they were to be used and held in individual plastic containers (30 mL, Maryland Plastics, Federalsburg, MD, USA).

### Treatment Preparation

To induce microbial growth for volatile and behavioral assays, microbial growth was promoted by tempering organic, hard winter wheat all obtained from a single farmer in Kansas to specific moistures through the addition of reverse osmosis, deionized water. To assess the baseline moisture level of the source grain, a grain moisture analyzer was used (DICKEY-john, GAC2100, Auburn, Illinois, USA). Source and control grain contained a moisture level of 10.8%. Every 7–9 d, from 21 May to 25 Jul 2018, two replicates of 200 g of organic wheat were tempered up to 12%, 15%, or 19% grain moisture levels by pipetting the appropriate amount of water, and thoroughly mixing grain. This represents the range of reasonable grain moisture levels for most commodities, as well as one just outside of that range (e.g. 19%), which mimics an insect hotspot or other control failure. Ambient fungal and microbial spores located on the grain itself formed the basis for microbial growth on the tempered grain. After tempering, batches of grain were incubated in an environmental chamber (30 °C, 60% RH, 14:10 L:D; Percival Instruments, Dallas County, IA, USA) for 9, 18, or 27 (± 2) d to promote microbial growth. As a negative control, we compared the grain moisture treatments to clean grain without the addition of extra water (i.e. untempered). Finally, for the maximally damaged grain by microbes mentioned for the near-infrared spectroscopy, grain was prepared as above and tempered to 19% grain moisture; the same amount of water was then added to the grain on a weekly basis for six weeks.

### Near-Infrared Spectroscopy of Grain

A multispectral sorting device was used to assess the proportion of grain kernels in treatments that were infested with fungi and other microbes, which manifested in color or reflectance changes (Pearson et al. 2013). Prior work has found that this sorter can correctly identify 90% of grain kernels damaged with the fungal disease, *Fusarium* head blight, from undamaged kernels (Pearson et al. 2013). The sorter used three visible and three near-infrared light-emitting diodes with peak emission wavelengths of 470 nm, 527 nm, 624 nm, 850 nm, 940 nm, and 1070 nm by rapidly blinking each LED (~ 12 kHz) in quick succession, and measuring the reflected light from the wheat kernels using a miniature 16 mm lens (V-4316-2.0, Marshall Electronics, Inc., CA, USA) as kernels dropped off a feeder chute at a 45° angle with a speed of 1.5 m/s. The device employed a microcontroller (ATmega328P, Atmel Corp., San Jose, CA) that directs the LED pulses, digitizes the analog signal from a photodiode (PDB-C171SM, Advanced Photonix, Inc., Ann Arbor, MI, USA), performs signal processing, and applies classifications. The device sorted grain at 20 kernels/s through the activation of an air valve that diverted the wheat kernels based on a prior calibration. Calibration of the machine occurred by training the machine to recognize damaged grain by using 200 fungal-damaged kernels and 200 undamaged kernels. Prior to data collection, we evaluated the accuracy of the sorter by calibrating the device based on training with the 12%, 15%, and 19% grain moisture incubated at 27 d, or for maximally infested grain (as described above) in comparison to clean grain held at 10.8% moisture in each case. The 19% grain moisture calibration was used for data collection based on its high accuracy (see results below), to obtain a conservative estimate of microbial damage, and to ensure consistency with incubation methodology compared to all other treatments. To evaluate damage, 100 g of grain, approximately 1,900 individual kernels, was sorted for each combination of grain moisture and incubation period, including the undamaged grain (control).

### Wind Tunnel Assay

**Fig. 1:**
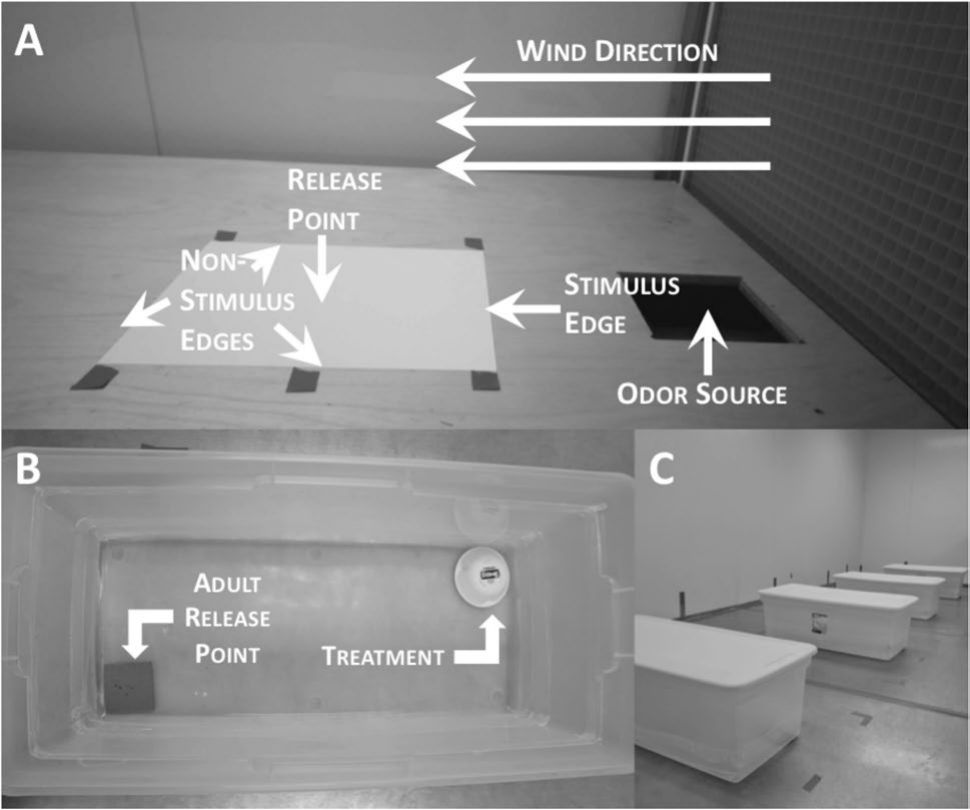
Experimental setup for **A** Wind tunnel assay, **B** each bin in the release-recapture assay with the cardboard slat release-point and the pitfall Dome Trap™, and **C** deployment of bins in release-recapture assay in a walk-in environmental chamber

A wind tunnel assay was used to better understand the taxis and attraction of *T. castaneum* and *R. dominica* to fungal volatiles from the grain*.* In the wind tunnel, air was pushed through a turbine (diameter: 36.5 cm), an activated carbon filter to purify the air, and two slatted-metal sieves (73 × 85 cm) to create a laminar airflow, with an average airspeed of 0.38 m/s. A total of 20 g of each odor treatment described above was placed 13.5 cm upwind of the stimulus edge of the test arena and 5 cm from the last sieve. Each odor treatment was held in a plastic petri dish (100 × 15 mm D:H). The adults were placed individually in the release point at the center of an arena consisting of a 21.6 × 27.9 cm sheet of paper. For all replicates, each adult remained walking in the wind tunnel. In each round of sampling, mixed-sex adult beetles were collected from their colonies using a sieve (No. 25, W.S Tyler Inc., Mentor, Ohio). Each adult was given a maximum of 2 min to respond, and the edge of the arena on which the adult exited was recorded as either the stimulus edge (upwind edge of the arena), or a non-stimulus edge (one of the other three edges) (Fig. 1A). In addition, the time to decision was recorded for each individual. Adults that did not respond within the timeframe were excluded from the final statistical analysis. Each adult was considered a replicate and was never used more than once. Odor flow from the petri dish to the release arena was confirmed with a smoke test. In total, there were *N* = 60 replicates per each species and treatment combination of grain moisture and incubation interval.

### Release-Recapture Trapping Assay

To assess how fungal volatiles may improve capture of *T. castaneum* and *R. dominica* adults in monitoring traps, a trapping assay was utilized (Fig. 1B). In each replicate, 20 mixed-sex adults were settled on clean cardboard slats (8 × 8 cm) and then placed in one corner of a plastic bin (86.3 × 30.5 × 39.4 cm L:H:W). In the opposite corner, diagonally across from the release point in the bin, a pitfall trap (Dome Trap™, Trécé, Inc., Adair, OK, USA; Campbell 2012) was deployed. The base was rough along the ascending edges to create traction for the beetles; and the crest was concave and smooth to prevent subsequent escapes. Semiochemical stimuli were placed within the concave portion of the trap. Each trap contained either no grain (negative control), or 5 g of one of the treatments: untempered grain (positive control), grain tempered to either 12%, 15%, or 19% grain moisture, or 950 µl of Trécé- produced Storgard™ Oil (Trécé, Inc.), a known semiochemical kairomone and food cue, which is a standard lure used in commercial facilities (Campbell 2012) and acted as the positive control. All grain in this study was either incubated for 9 d, 18 d, or 27 d, and trapping assays were initiated on the same days as the wind tunnel assays described above. The bins were located in a large (5 × 6 × 2 m, L:W:H) walk-in environmental chamber (Percival Instruments, Dallas County, IA, USA) under constant conditions (25 °C, 65% RH, 14:10 L:D). The adults were given 24 h to respond to the stimuli. Afterwards, the traps were collected, the adults were sieved (No. 10, W.S Tyler Inc., Mentor, OH), and the number of beetles in each trap was recorded. On a given round of release, two replicates of each treatment listed above were performed simultaneously, for a total of 12 bins set up containing 240 adults total. In total, there were *N* = 8 replicates per species and combination of treatment and incubation, including the controls.

### Volatile Collection

Headspace collection was performed in order to determine the volatiles released by the various moisture treatments (no grain as a negative control, or 20 g of untempered grain control, or grain tempered to 12%, 15%, or 19%) and incubated at different intervals (9 d, 18 d, 27 d). First, central air was scrubbed using an activated charcoal filter, then pushed through the remaining apparatus. The airflow was restricted to 1 L/min using a flow meter (Volatile Collection Systems, Gainesville, FL, USA) placed directly prior to the sample collection in headspace chambers (10.2 × 12.7 cm D:H) with an inlet for air and an outlet for a volatile collection trap (VCT). The headspace volatiles from the grain were collected for 3 h onto a VCT consisting of a drip tip borosilicate glass tube packed with 20 mg of absorbent Porapak-Q™ (Volatile Collection Systems, Gainesville, FL, USA) to adsorb volatiles with a stainless steel screen (No. 316) on one side, and held in place with a borosilicate glass wool plug followed by a PTFE Teflon compression seal. The volatiles on the traps were eluted with 300 µl of dichloromethane (Millipore, Billerica, MA, USA) by pushing the solvent through with inert nitrogen gas into 2-mL glass vials with caps containing Teflon-lined septa and then stored at −20 °C until analysis. Volatile collection traps were reused after they were washed three times with 700 µl of dichloromethane. Prior to analysis, tetradecane (190.5 ng, 99% purity, GC analytical grade, Millipore, Billerica, MA, USA) was added as an internal standard.. A total of *N* = 4–6 replicate collections were done for each combination of moisture and incubation treatment.

### Gas Chromatography Coupled with Mass Spectrometry

All headspace collection sample extracts were run on an Agilent 7890B gas chromatograph (GC) (Agilent Technologies, Inc., Santa Clara, CA, USA). equiped with an Agilent Durabond HP-5 column (30 m length, 0.250 mm diameter and 0.25 μm film thickness) with helium as carrier gas at a constant 1.2 mL/min flow and 40 cm/s velocity, which was coupled with a single-quadrupole Agilent 5997B mass spectrometer (MS). Samples were injected with an autosampler in split mode split (15:1 ratio with a split flow rate of 18 ml/min).The compounds were separated by auto-injecting 1 μl of each sample in split mode (250 °C) with flow was split in a 15:1 ratio with a split flow rate of 18 ml/min. The oven temperature was programmed at 40 °C for 1 min followed by 10 °C/min increase to 300 °C and then held for 26.5 min. After a solvent delay of 3 min, mass ranges between 50 and 550 atomic mass units were scanned. Compounds were tentatively identified by comparison of spectral data with those from the NIST 17 library and by GC retention index (Adams 2009). Compound peak areas relative to that of the internal standard were used to calculate the emission rates (ng/g of grain/h of collection).

### Microbial Community Characterization

To characterize the microbial community on the grain, the microbes were first cultured on potato dextrose agarose (PDA) petri dishes (100 × 10 mm). This was performed by subsampling 5 g of grain that had been incubated for 27 d at 18% grain moisture. Each subsample was thoroughly agitated for 1 min, then four wheat kernels were placed in one of each of the quadrants of a petri dish with PDA in a biosafety cabinet (Labconco, Kansas City, MO, USA). This was done for three separate replicate dates of grain incubation and tempering. Thus, a total of 12 wheat kernels were initially cultured for 10 d in an incubator (IMC18, Heratherm, ThermoScientific, Waltham, MA, USA) at room temperature (approx 23 °C). For each dish, every unique microbial morphotype was streaked onto a new PDA petri dish, and allowed to incubate for a further 10 d under the same conditions. At the end, 200 mg of fungal material was excised and used for DNA isolation and sequencing below. A total of *N* = 25 excisions was taken.

### Microbial Community DNA Isolation and Sequencing

Excised fungi were placed into 1.5-ml microcentrifuge tubes and DNA was extracted using the Quick-DNA Fecal/Soil Microbe Miniprep Kit (D6010, Zymo Research, Irvine, CA, USA). The ITS region of the fungal DNA was amplified using polymerase chain reaction (PCR), using primers that consisted of ITS4 5’-TCCTCCGCTTATTGATATGC-3ʹ and ITS5 5ʹ-GGAAGTAAAAGTCGTAACAAGG-3ʹ (White et al. 1990). In each reaction, 1 μl of each of extracted DNA and primer (10 μM), 9.5 μl of nuclease free water, and 12.5 μl of master mix containing 50 units/ml of Taq DNA polymerase (Hot Start Taq 2X Master Mix, Promega, Madison, WI, USA) were combined in a proprietary reaction buffer (pH 8.5). Briefly, the PCR program consisted of 2 min of initial denaturation at 95 °C, followed by 30 cycles of 95 °C for 30 s, 55 °C for 1 min, and 72 °C for 1.5 min. Afterwards, a final extension was performed at 72 °C for 5 min, then PCR products were held chilled at 4 °C. To clean up the samples, 5 μl of PCR products were mixed with 2 μl of ExoSAP-it (ThermoFisher Scientific, Waltham, MA, USA), then placed in the thermal cycler at 37 °C for 15 min, and ramped to 80 °C for a further 15 min. Finally, the amplicons were sent for bidirectional sequencing on an ABI 3730XL instrument (Eurofins Scientific, Brussels, Belgium), and the resulting sequences were quality-filtered and aligned using Sequencher (v. 5.4.6, Gene Codes, Ann Arbor, MI, USA). The consensus sequences were searched against NCBI’s nucleotide database (nt) using the BLASTn algorithm (Altschul et al. 1997). In order to circumvent taxonomic misassignments, the consensus sequences were also checked against Michigan State’s Ribosomal Database Project (RDP) that searches the UNITE Database (Wang et al. 2007). The consensus sequences were submitted to GenBank and under the accession numbers MT883436–MT883460.

### Sensory Panel Evaluation of Grain

In order to confirm that the volatiles coming off of the grain were indeed of microbial in origin and not simply grain volatiles that have also been ascribed a dual purpose as MVOC’s from the literature, an informal sensory panel was convened from three volunteer technicians at the USDA Center for Grain and Animal Health Research. Each volunteer was familiar with unadulterated and spoiled grain from extensive experience handling insect colonies on grain. Each volunteer was given the opportunity to smell the bouquet of *N* = 4 replicates of each treatment combination of incubation period (9d, 18 d, 27 d) and grain moisture (ctrl, 12%, 15%, 18%) listed above and was asked to report the odor as “grain-like”, “spoiled”, or “other”. Exactly 5 g of each treatment was placed in a 3-oz capacity plastic container, were covered with lids, and then randomly assigned a location on tray with the identity of the treatment known only to an impartial third observer not participating in the study. Volunteers then sequentially sniffed each treatment, leaving 1 min between each sniff in order to allow a recovery time to cleanse the palette prior to additional tests. On a given day, a single person smelled 16 samples.

### Statistical Analysis

Differences in fungal damage from near-infrared spectroscopy were analyzed by comparing the proportion of damaged grain (by weight) of 100 g among grain moisture treatments, and within a grain moisture treatment among incubation intervals using χ^2^-tests relative to the null hypothesis that damage was equal between treatments. A Bonferroni correction to the α-threshold was used to control for the family-wise error rate.

To analyze the data from the wind tunnel assay, a generalized linear model based on a binomial distribution was employed. In particular, adult beetles that exited on the stimulus (coded as 1) or non-stimulus edge (coded as 0) of the arena was used as the response variable. The two explanatory variables included in the model were the grain moisture level (control, 12%, 15%, or 19%) and incubation interval (9 d, 18 d, or 29 d), as well as their interaction. The model was evaluated for overdispersion, which was not found to be an issue. Log-likelihood tests for significance were conducted based on a χ^2^-distribution. Upon a significant result from the model, generalized linear model multiple comparisons was conducted using the function *glht* in the *multcomp* R package (Hothorn 2017) which implements Tukey’s HSD for generalized linear models. For this and all other analyses, R v.4.0.3 was used (R Core Team 2021), and α = 0.05, except where otherwise noted.

Differences in the time (seconds) to decision for the wind tunnel were analyzed with a 2-way analysis of variance (ANOVA). The model structure was of the same form as the one in the preceding paragraph. After inspection of residuals, the data conformed to the assumptions of normality and homogenous variances, and thus no transformation was necessary. After a significant result from the overall model, Tukey’s HSD was used for pairwise comparisons.

For the trapping assay, data were analyzed with a two-way ANOVA, using the number of adult *T. castaneum* and *R. dominica* recaptured in traps as response variables. The two explanatory variables included in the model were the lure treatment (no grain, untempered grain, grain tempered to 12%, 15%, or 19%, or Trécé Storgard Oil) and grain incubation interval (9 d, 18 d, or 27 d), as well as their interaction. The data conformed to the assumptions of normality and homogenous variances, and thus no transformation was necessary. After a significant result from the overall model, Tukey’s HSD was used for pairwise comparisons.

To analyze the volatiles collected from different grain moisture and incubation treatments, raw peak areas were extracted from the gas chromatograms using MSD ChemStation v2.00 software (Agilent Technologies, Inc., Santa Clara, CA, USA). Compounds were aligned between representative samples from each treatment based on retention time and quantified using the internal standard as above. Background volatiles found in the negative control without grain were discarded from the other samples, because these represent transient background volatiles in the general vicinity of headspace collection but are not informative of differences among the treatments. In addition, headspace compounds found in only two samples were discarded, because these represent transient emissions not representative of differences among treatments. Using the emissions of individual volatiles, Bray–Curtis dissimilarities were calculated in a pairwise fashion between each headspace sample, and non-metric multi-dimensional scaling (NMDS) was used to visualize the differences in volatile emissions among treatments. A total of *N* = 1000 permutations was used for the ordination procedure. Stress values for the NMDS procedure were < 0.1, indicating that good interpretation was possible. An analysis of similarity (ANOSIM) was used to determine significant differences for headspace volatiles among levels within grain moisture (negative control, positive control, 12%, 15%, 19%), and by incubation interval (9 d, 18 d, 27 d). A total of *n* = 1000 permutations was performed for the test. For all multivariate statistics, the *vegan* package was used in R (Oksanen et al. 2019).

To analyze the data from the sensory panel evaluation, a generalized linear model based on a binomial model was used. The response variable included whether samples were spoiled (yes/no), with incubation period, grain moisture, and their interaction used as fixed explanatory variables. Overdispersion was checked and found not to be an issue with the model. Multiple comparisons employed chi-squared tests with Bonferroni correction to the alpha-threshold.

## Result

### Near-Infrared Spectroscopy

**Fig. 2:**
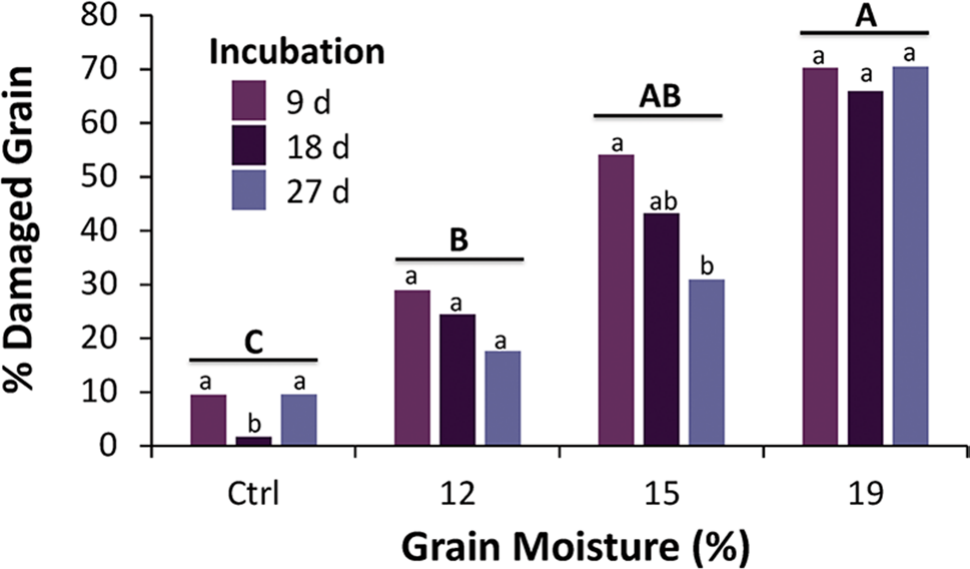
The percent of damaged wheat grain kernels from 100 g per incubation interval and treatment based on sorting by a near-infrared device. Lower case letters are comparisons within a moisture treatment, while upper case letters are comparisons among grain moisture treatments. Bars with shared letters are not significantly different from each other (χ^2^-test, Bonferroni correction)

Overall, the sorter was able to accurately sort 95% of the sound kernels from the damaged grain and had an error rate of 13% for sorting the damaged grain from the sound kernels. In ascertaining fungal growth, the percentage of damaged kernels present differed significantly among all grain moisture treatments (pairwise χ^2^ – tests, *P* < 0.05; Fig. 2). In general, as the moisture increased in the grain from the control to 19%, the percent of damaged grains nearly doubled at each level. The incubation intervals did not significantly affect the amount of damaged grain (χ^2^-tests, Bonferroni correction; Fig. 2). Overall, the lowest percent of damaged grain was in the untempered control grain, which was almost 10 times less than the 19% grain moisture treatment.

### Wind Tunnel Assay

**Fig. 3:**
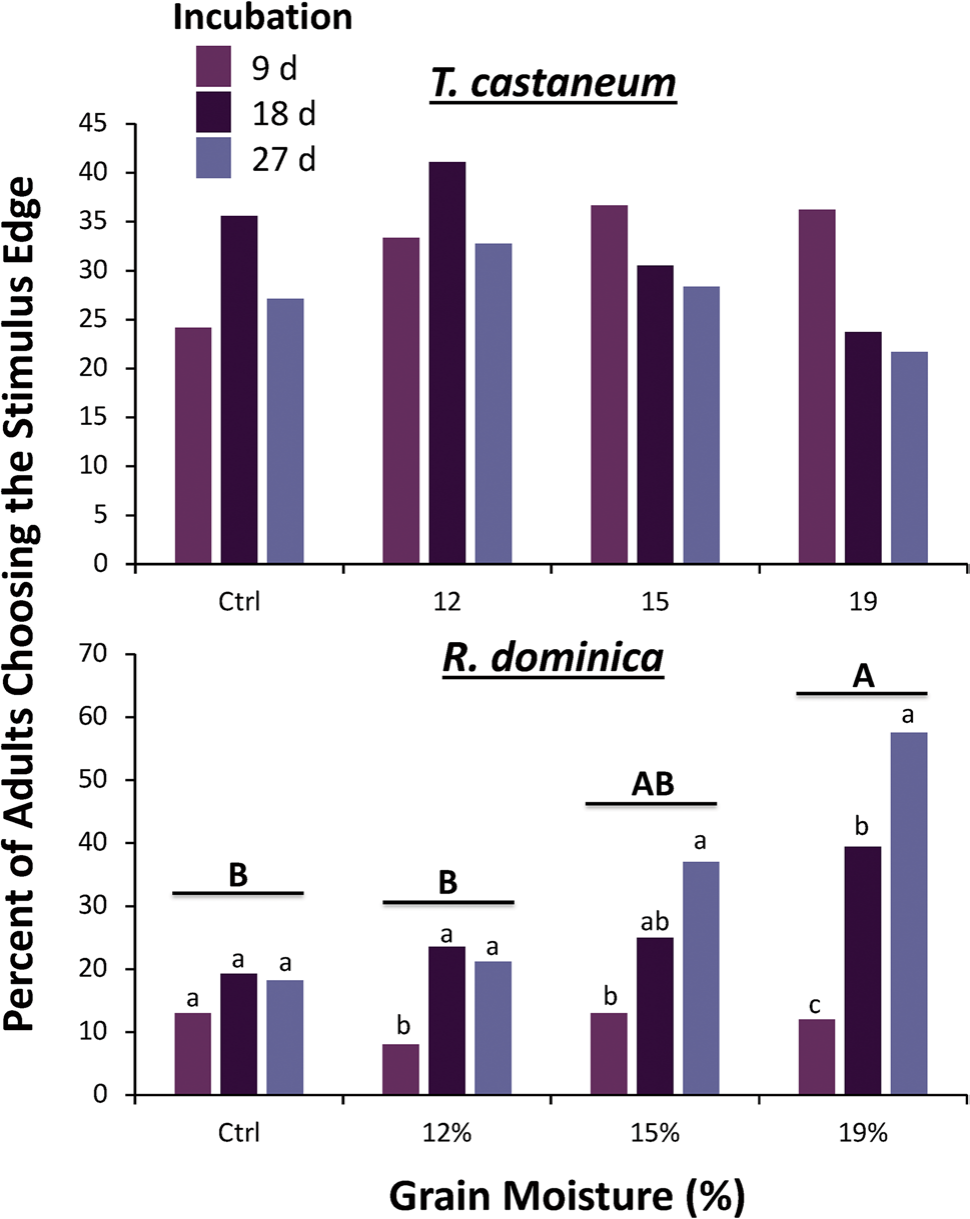
The percent of **A** *Tribolium castaneum* (*N* = 60 replicates per bar) and **B** *Rhyzopertha dominica* (*N* = 60 replicates per bar) adults that chose the stimulus edge in response to grain moisture treatments and incubation intervals in a wind tunnel assay. Lower case letters are comparisons within a moisture treatment. Bars with shared letters are not significantly different from each other (χ^2^-test, Bonferroni correction). No letters are displayed for *T. castaneum*, because statistical tests for the overall model were not significant

Grain moisture did not significantly affect the proportion of *T. castaneum* adults choosing the upwind stimulus edge of the arena (χ^2^ = 4.33; df = 3; *P* = 0.63; Fig. 3). The total percentage of adults choosing the stimulus side ranged from 28% to 37% in the various grain moisture treatments. In addition, we found that there was no significant difference in the proportion of *T. castaneum* choosing the stimulus edge of the arena at different incubation intervals (χ^2^ = 1.59; df = 2; *P* = 0.21; Fig. 3), which ranged from 27% to 44%. The interaction between grain moisture and incubation time also had no significant effect on the behavior of adult beetles (χ^2^ = 6.32; df = 6; *P* = 0.39). There was only an 18.7% difference between the treatments eliciting the lowest and highest percentages of adults that chose the stimulus edge. Likewise, neither the grain moisture (*F* = 0.09; df = 3, 698; *P* = 0.96) nor the incubation interval (*F* = 0.13; df = 2, 698; *P* = 0.72) significantly affected *T. castaneum* adult decision time. There was also no significant grain moisture by incubation interval interaction on the time it took *T. castaneum* adults to reach an edge (*F* = 0.49; df = 6, 698; *P* = 0.69).

However, we found that there was a significant difference in the attraction of *R. dominica* to the various incubation levels (χ^2^ = 5.86; df = 2; *P* < 0.05; Fig. 3), with 1.6-fold greater attraction to grain incubated for 27 d than 9 d. In contrast to *T. castaneum*, we found that the grain moisture treatments had a significant effect on the percentage of *R. dominica* choosing the stimulus edge (χ^2^ = 15.1; df = 3; *P* < 0.01; Fig. 3). The total percentage of adults that chose the stimulus edge was two-fold greater in the 19% grain moisture treatment compared to the grain tempered to 12%. There was no significant interaction found between grain moisture treatments and incubation intervals (χ^2^ = 3.65; df = 3; *P* = 0.30; Fig. 3).

However, the grain moisture did not significantly affect the time required for *R. dominica* adults to make decisions (*F* = 0.93; df = 3, 466; *P* = 0.43). In addition, the incubation interval did not significantly affect adult decision time (*F* = 3.04; df = 2, 466; *P* = 0.08), with the time to decision ranging from 58 to 63 s. There was no significant interaction between grain moisture and incubation interval on the time it took *R. dominica* adults to make decisions (*F* = 0.99; df = 6, 466; *P* = 0.40).

### Release-Recapture Trapping Assay

**Fig. 4:**
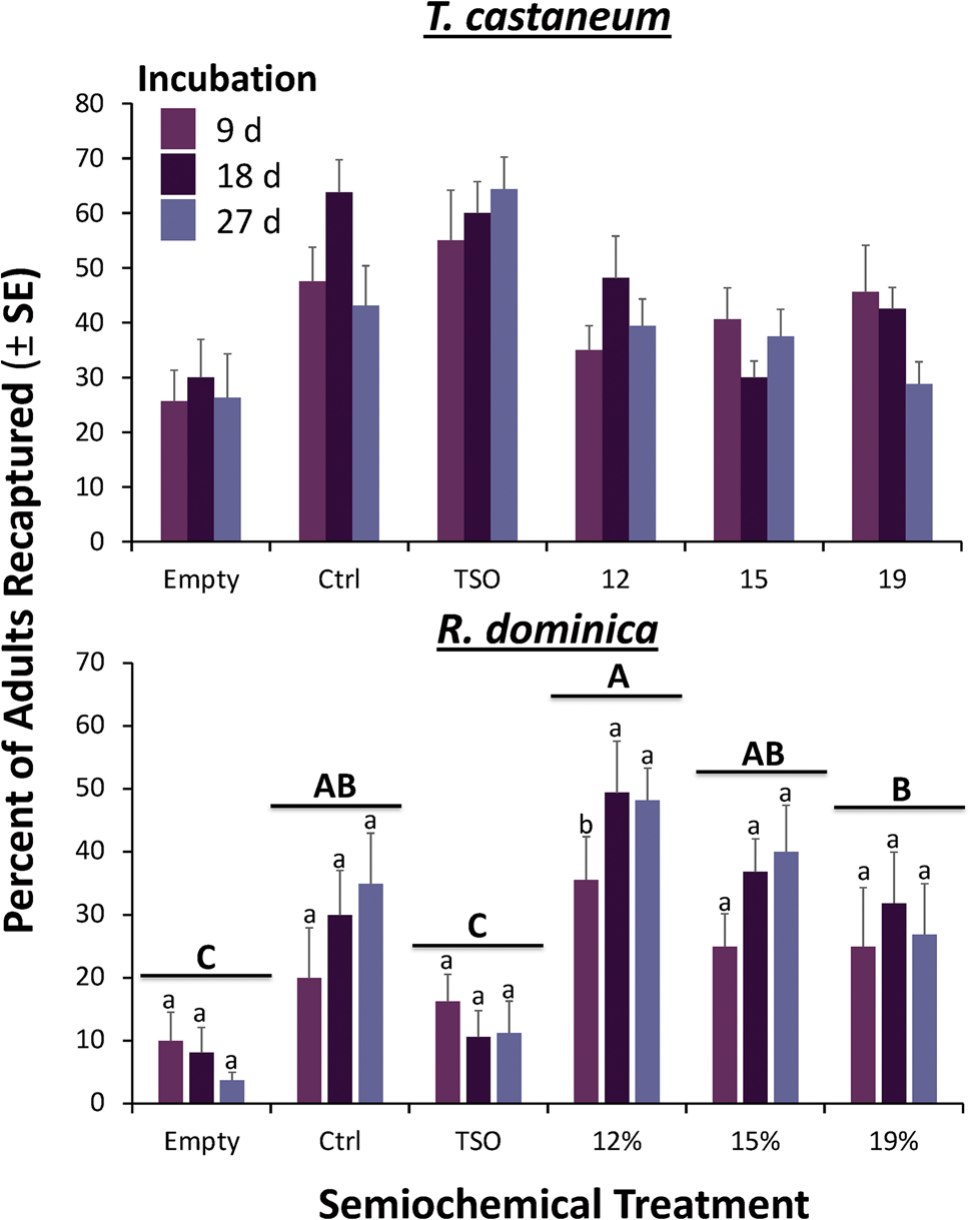
The percent (± SE) of *Tribolium castaneum* (*N* = 8 replicates per bar) and *Rhyzopertha dominica* (*N* = 8 replicates per bar) adults recaptured in pitfall traps which contained various semiochemical treatments. Empty: unbaited pitfall trap, Ctrl: positive control of untempered grain, TSO: Trécé Storgard oil, 12, 15, 19: grain moisture treatments. For both species, the interaction between semiochemical treatments and incubation times was not significant. Bars with shared letters are not significantly different from each other (Tukey HSD, α = 0.05). No letters are displayed for *T. castaneum*, because statistical tests for the overall model were not significant

In the release-recapture assay, across all treatments and incubation levels, 42.4% of *T. castaneum* adults released were recaptured, indicating a sufficient sample size to form conclusions. We found that there was no significant difference between grain moisture treatments on the recapture of *T. castaneum* adults (*F* = 0.43; df = 5; *P* = 0.83; Fig. 4). The largest difference in the percentage of adults recaptured in an incubation interval was 38.1% between the negative control and the commercial TSO (Storgard) lures at 27d. The number of adults recaptured was not significantly different among incubation intervals (*F* = 0.063; df = 2; *P* = 0.80; Fig. 4), which ranged between 27% and 44% across moisture treatments. Finally, the interaction between grain moisture and incubation interval did not have a significant effect on the recapture of adults (*F* = 0.69; df = 5; *P* = 0.63; Fig. 4).

Similar to *T. castaneum*, across all treatment combinations, 28% of all released *R. dominica* adults were recaptured in the pitfall traps, indicating a sufficient sample size to develop conclusions. However, in contrast to *T. castaneum*, the semiochemical lures had a significant effect on the capture of *R. dominica* adults compared to empty traps (*F* = 4.31; df = 5; *P* = 0.001; Fig. 4). *Rhyzopertha dominica* showed elevated capture in pitfall traps with moderate fungal growth in grain tempered to 12% (Tukey HSD, Fig. 4). The total percentage of adults captured in the traps with 12% grain moisture was 3.6 times greater than empty traps. The incubation interval did not significantly affect the number of *R. dominica* adults in traps (*F* = 0.22; df = 2; *P* = 0.64; Fig. 4).

### Volatile Collections

**Fig. 5:**
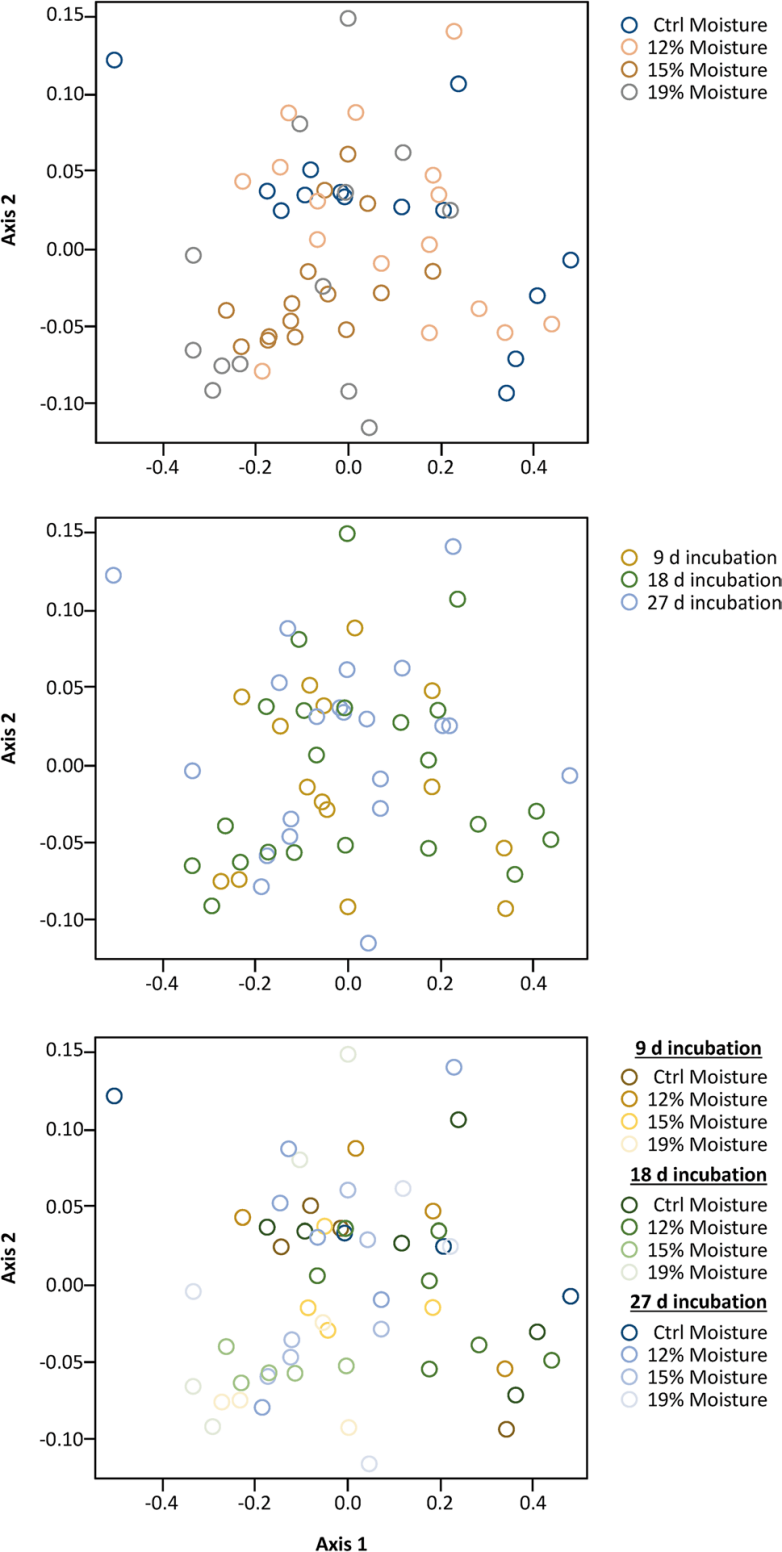
Non-metric multidimensional scaling (NMDS) ordination of headspace volatiles from grain samples that were not tempered (positive control, grain moisture ≈ 10.8%), or tempered to 12%, 15%, or 19% grain moisture and incubated for 9 d, 18 d, or 27 d (*N* = 4–6 replicates per treatment combination). Shading of samples (circles) is based on **A** Grain moisture, **B** Incubation period, or **C** Their interaction. Ordination is based on pairwise Bray–Curtis similarities calculated among samples. Calculated stress is < 0.06 after *n* = 1000 permutations, indicating interpretation is possible

The GC–MS analyses of volatiles collected showed that, while there was significant overlap in volatile emissions by treatments, there was some significant differentiation. For example, grain moisture had a small but significant impact on the composition of volatiles emitted from the treatments (R = 0.116; Perm = 1000; *P* < 0.001; Fig. 5). However, there was no significant difference among the volatiles emitted from grain at different incubation intervals (R = −0.033; Perm = 1000; *P* = 0.93; Fig. 5). While the headspace from the different incubation intervals were equally complex (χ^2^ = 0.09, df = 2, *P* = 0.95), each with about 15 compounds, the grain tempered to 19% moisture was more complex (χ^2^ = 4.15, df = 2, *P* < 0.05), with 1.6-fold more compounds than the positive control consisting of unmanipulated grain (Supplementary Table 1). Tempered grain appears to be enriched in 2-methyl- and 3-methyl-1-butanol compared to the positive control grain as well as other assorted alkane and alkene alcohols (Supplementary Table 1).

### Microbial Community Characterization

In all, 25 samples were sequenced. Most of the fungi identified belonged to the genus *Alternaria* (68%), followed by *Aspergillus* (16%), *Diplodia* (8%), *Penicillium* (4%), and *Epicoccum* (4%) (Table 1). In total, nine species were identified from each of these genera, with the most species rich genus being *Alternaria*.

**Table 1:** Microbial community characterization from 300 g wheat grain samples incubated for 27 d after tempering to 19% grain moisture

Genus	Species	No. of samples	Percent of samples
*Alternaria*		17	68
	*alternata*	6	24
	*arborescens*	1	4
	*infectoria*	6	24
	*tenuissima*	4	16
	*subtotal*	17	68
*Aspergillus*			
	*tubingensis*	3	12
	*niger*	1	4
	*subtotal*	4	16
*Diplodia*		2	8
	*seriata*	2	8
*Penicillium*		1	4
	*rubens*	1	4
*Epicoccum*		1	4
	*layuense*	1	4
Total		25	100

#### Sensory Panel Evaluation

**Fig. 6:**
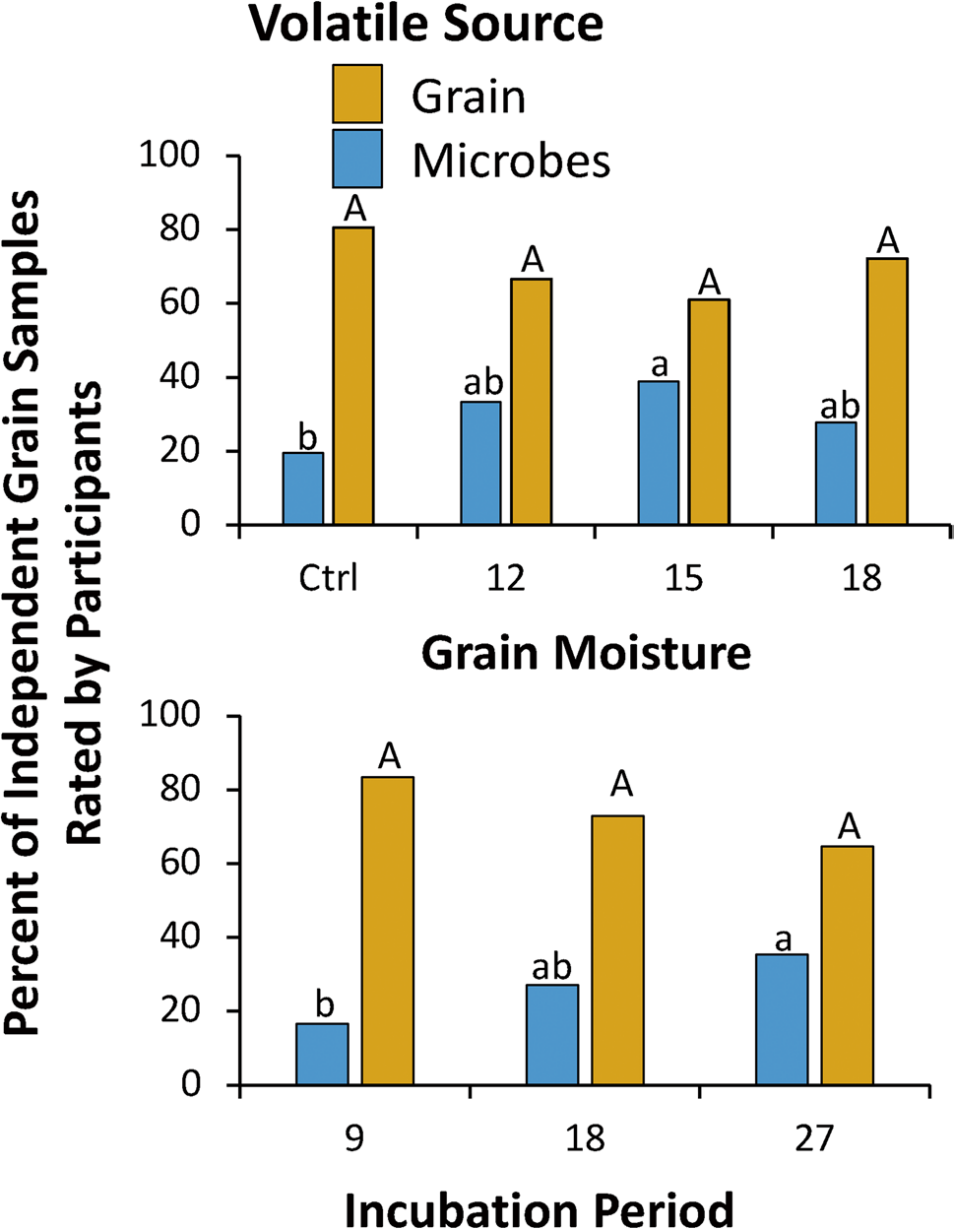
Result of the sensory panel evaluation conducted by three technical personnel at USDA Center for Grain and Animal Health Research confirming source of volatiles as originating from microbial contamination (blue bars) or grain (orange bars). Each bar represents the overall percentage of *N* = 36 (top panel) or *N* = 48 (bottom panel) independent assessments conducted by three volunteers in a series of four replicates. Capitalized letters represent comparisons among grain-odor bars, while lower case letters represent comparisons among microbial odor bars. Bars with shared letters are not significantly different from each other (χ^2^-test, Bonferroni correction)

Both the grain moisture (χ^2^ = 7.82; df = 3; *P* < 0.05) and incubation period (χ^2^ = 6.70; df = 2; *P* < 0.05) had a significant effect on the percentage of samples perceived as originating from a microbial source (Fig. 6). However, the interaction between the two factors did not significantly affect perception of microbial contamination (χ^2^ = 8.37; df = 6; *P* = 0.21). Other unprompted comments by the volunteers to describe the microbially-contaminated grain included: “musty”, “malodorous”, “moldy”, “sour”, “spoiled”, and “pungent”. Unprompted comments describing volatiles primarily from the unspoiled grain included: “grain-like”, “moist”, and “wet grain”.

## Discussion

In this study, we have demonstrated that the primary pest, *R. dominica*, is attracted to MVOC’s found in tempered and incubated grain samples, both in the wind tunnel and in a release-recapture experiment. The sensory panel confirmed that perception of microbial volatiles was highest among the treatments found to be most attractive to *R. dominica*. This was counter to our original hypothesis where we thought fungal cues may be more important to the secondary pest, *T. castaneum*. To our knowledge, this is the first study to document attraction of *R. dominica* to MVOC’s. Previous work reported that the presence of other stimuli such as aggregation pheromones (Dissanayaka et al. 2020; Edde et al. 2005; Leos-Martinez et al. 1987) or presence of conspecifics (Cordeiro et al. 2019) can affect the orientation of *R. dominica*. Furthermore, both sexes of *R. dominica* respond to plant volatiles, with the volatiles from wheat seeds being the most preferred (Edde and Phillips 2006). Additionally, when ethanol was added to the *R. dominica* aggregation pheromone (dominicalures 1 & 2) in funnel traps, there was a significant increase in field captures, but no increase was observed when a variety of essential oils or green leaf volatiles were combined with the pheromone (Edde et al. 2011). Bashir et al. (2001) found a synergistic attraction by both sexes of *R. dominica* to maize volatiles and conspecific cues. By contrast, Nguyen et al. (2008) found no evidence that walking *R. dominica* use cereal volatiles to locate grain. While the interactions between *R. dominica* with plant volatiles, grain volatiles, and conspecific cues has been extensively explored, its interaction with the microbial community has been neglected.

Contrary to our initial hypothesis, the secondary feeder, *T. castaneum*, did not seem to use microbial cues, while *R. dominica* did in fact use them and apparently to a much more generalized extent, given the diverse assemblage of microbes that may have been present on the grain. One potential evolutionary rationale for the use of microbial cues by *R. dominica* is in its historical use of woody hosts and animal caches as food sources. Microbial and/or fungal colonization of grain or wood may have been historically helpful for the successful feeding and oviposition by *R. dominica,* because it weakens the grain kernel’s bran (e.g., outer shell) in the case of stored products or weakens the bark in the case of woody natural hosts, allowing individuals of a variety of taxa easier access (Biedermann et al. 2019). *Rhyzopertha dominica* is a bostrichid, a family of wood-boring beetles that also includes another important stored product pest, *Prostephanus truncates* Horn, which has a documented affinity for stressed or already girdled trees in natural landscapes (Quellhorst et al. 2021). Indeed, *R. dominica* is readily captured far from food facilities among native habitats (Jia et al. 2008; Mahroof et al. 2010), suggesting that host switching may occur. While foraging, *R. dominica* may use MVOC’s in enclosed environments to navigate, their use of these cues in the field should be confirmed. Thus, our study helps to highlight the importance of microbial cues for foraging of some stored product insects, depending on their life history. In addition, our results suggest that future work elucidating the interaction of *R. dominica* with the microbial community may be worthwhile.

Unlike *R. dominica*, we did not find elevated attraction to MVOC’s by the secondary pest, *T. castaneum*. As a secondary pest, *T. castaneum* is not able to access resources in intact grain (Hagstrum and Subramanyam 2006). As a consequence, it is especially surprising that *T. castaneum* did not respond to the MVOC’s in this study, because colonization by a low number of microbes may be able to weaken a kernel sufficiently so that it could be used by *T. castaneum*. Indeed, prior work has documented that, after damage by a primary pest, wheat kernels were more attractive to *T. castaneum* than intact or mechanically-damaged kernels (Trematerra et al. 2000), suggesting that invasion of a grain mass by species like *R. dominica* and *Sitophilus oryzae* (L.) (Coleoptera: Curculionidae) may facilitate colonization of *T. castaneum*. Thus grain conditioned or infested with *Sitophilus zeamais* (Motschulsky) (Coleoptera: Curculionidae) was in fact found to be preferred by *T. castaneum* in olfactometer assays (Trematerra and Ianiro 2015). It is clear that *T. castaneum* then may follow primary pests to access nutrients in intact grain more easily, but this makes the lack of response to cues from another pest that could open up an otherwise inaccessible resource even more unexpected. The varying response by species with different life histories to MVOC’s further highlights the importance of understanding the ecological interactions between insects and fungi in the postharvest environment.

The lack of response by *T. castaneum* may partly be explained by the setup of the current study. The MVOC’s in this study originated from any fungi that were already present on the grain at the beginning of the experiment. In fact, we confirmed that there was a diverse community of fungi that had colonized the grain, with *Alternaria* sp. and *Aspergillus* sp. as the most common groups present. In total, we isolated nine species of fungi. Multiple species of microbes in the grain may have been emitting behaviorally antagonistic volatiles that could have produced no net change in attraction. Varying blends of volatiles are produced by specific fungal species that colonize grain (Magan and Evans 2000). This has implications for attraction and preference by the stored product insect community. For example, *Tenebrio molitor* L. (Coleoptera: Tenebrionidae) avoided grain infested with *Fusarium avanaceum* or *Beauveria bassiana*, but were attracted to grain with *F. proliferatum*, *F. poae*, or *F. culmorum* (Guo et al. 2014). Ahmad et al. (2012) found moldy cotton lint to be attractive to *T. castaneum*; in follow-up tests, the authors cultured five unidentified fungal strains isolated from the cotton and found conspecifics were attracted to one of the strains, repulsed by another, whereas the other three had no behavioral effects. Thus, depending on the relative abundance of microbial species in the grain, and the strength of behavioral response by the insect community, the signal-to-noise ratio may be too high to detect a behavioral change. If true, this also suggests that *R. dominica* is potentially broadly attracted to a greater range of MVOC’s than *T. castaneum.* While Rering et al. (2020) found that response to microbial monocultures and microbial assemblages were similar by the European honey bee, *Apis mellifera*, the generalization of this finding is currently unknown, and may vary in other systems. Future research should evaluate behavioral response to microbial monocultures found from grain in the postharvest environment to tease apart which species might be producing volatiles of behavioral relevance to stored product insects.

In this study, we tempered grain with free water, a common milling technique, to elevate grain moisture between 12% and 19%, which roughly conforms to the practical range of tempering used in the wheat milling industry (12–17%) to facilitate separation of the bran and germ from white flour (Carter et al. 2015). While the incubation periods in this study were longer than those typically found at food facilities, it did offer microbes a chance for proliferation, and microbial growth was confirmed as a source of volatiles over grain by the novice sensory panel in this study. Another approach to allowing microbial growth in the grain would have been conditioning grain in salt chambers to equilibrate to atmospheric moisture conditions over time, but this would have required significantly longer periods of time; time could not have been varied independently. Prior work using this methodology found the primary fungal species on maize after 348 and 751 d consisted of 20 species, including *Acremonium zeae*, *A. flavus*, *Penicillium pinophilum*, and others (Wicklow et al. 1998).

Prior literature has described a variety of aldehydes, alcohols, ketones, alkanes, and alkenes that are characteristic of wheat (Mattiolo et al. 2017). Common fungal volatiles include 1-octen-3-ol, 3-octanol, 3-methyl-butanol, and 3-octanol (Herrera et al. 2015; Morath et al. 2012; Ponce et al. 2021), and some of these were in the various tempered wheat treatments. For example, in the 19% moisture treatment incubated for 18 d or 27 d, 1-octen-3-ol was a significant constituent (Supplementary Table 1). The fact that certain fungal metabolites were found in some tempered treatments, but not all, suggests that the fungal community may not have been identical among tempered treatments. Ponce et al. (2021) recently found that the behavioral response by the stored product insect community to MVOC’s may be context and species dependent. Knowing which of the volatiles in these complex headspaces are perceived by stored product insects may be done in the future by gas chromatography coupled with electroantennography to help develop a list of target compounds for future work. This will help prioritize which specific compounds to test for behavioral experiments in order to develop behaviorally relevant semiochemicals to support postharvest protection of commodities.

Finally, interactions between the insect and microbial communities are important in the postharvest environment, and may have important implications for grain quality (Quellhorst et al. 2021). Even when there is no visible mold growth, there may imperceptible (e.g., to human eyes) but consistent changes in the grain in the near infrared, confirmed by sequencing microbes on the grain, and emission of off-odors that are deleterious to grain quality, as in this study. Changes in volatile profiles have been used as indicators of food and feed spoilage (Schnürer et al. 1999). Moreover, prior work has found that insects and microbes may alter grain environments in ways that are mutually beneficial (Ponce et al. 2021), for example by altering the microclimate in grain masses in ways that enhance the fitness of the interacting partner. Often, the management of insects and microbes is considered separately at food facilities, but our study highlights that they may significantly affect each other and may need to be managed simultaneously.

## Supplementary Information

Below is the link to the electronic supplementary material.

Supplementary file1 (XLSX 32 kb)

## Data Availability

The datasets presented in this study can be found in online repositories. The names of the repository/repositories and accession number(s) can be found at: Van Winkle, Taylor; Ponce, Marco; Quellhorst, Hannah E.; Bruce, Alexander; Albin, Chloe, E.; Kim, Tania N.; Zhu, Kun Yan; Morrison, William R.. Dataset for “Microbial volatile organic compounds mediate attraction by a primary but not secondary stored product insect pest in wheat”. Ag Data Commons. https://data.nal.usda.gov/dataset/dataset-microbial-volatile-organic-compounds-mediate-attraction-primary-not-secondary-stored-product-insect-pest-wheat. Accessed 2021-02-19.

## Code Availability

Code is available upon request to corresponding author.
